# Plasticity of Cyanobacterial Thylakoid Microdomains Under Variable Light Conditions

**DOI:** 10.3389/fpls.2020.586543

**Published:** 2020-11-12

**Authors:** Myriam Canonico, Grzegorz Konert, Radek Kaňa

**Affiliations:** ^1^Institute of Microbiology, CAS, Centrum Algatech, Třeboň, Czechia; ^2^Faculty of Science, University of South Bohemia, České Budějovice, Czechia

**Keywords:** photosynthesis, thylakoid membrane, microdomains and rafts, membrane organization, cyanobacteria, phenotypic heterogeneity, photosystems, phycobilisomes decoupling

## Abstract

Photosynthetic light reactions proceed in thylakoid membranes (TMs) due to the activity of pigment–protein complexes. These complexes are heterogeneously organized into granal/stromal thylakoids (in plants) or into recently identified cyanobacterial microdomains (MDs). MDs are characterized by specific ratios of photosystem I (PSI), photosystem II (PSII), and phycobilisomes (PBS) and they are visible as sub-micrometer sized areas with different fluorescence ratios. In this report, the process of long-term plasticity in cyanobacterial thylakoid MDs has been explored under variable growth light conditions using *Synechocystis* sp. *PCC6803* expressing YFP tagged PSI. TM organization into MDs has been observed for all categorized shapes of cells independently of their stage in cell cycle. The heterogeneous PSI, PSII, and PBS thylakoid areas were also identified under two types of growth conditions: at continuous light (CL) and at light-dark (L-D) cycle. The acclimation from CL to L-D cycle changed spatial distribution of photosystems, in particular PSI became more evenly distributed in thylakoids under L-D cycle. The process of the spatial PSI (and partially also PSII) redistribution required 1 week and was accompanied by temporal appearance of PBS decoupling probably caused by the re-organization of photosystems. The overall acclimation we observed was defined as TM plasticity as it resembles higher plants grana/stroma reorganization at variable growth light conditions. In addition, we observed large cell to cell variability in the actual MDs organization. It leads us to suggest that the plasticity, and cell to cell variability in MDs could be a manifestation of phenotypic heterogeneity, a recently broadly discussed phenomenon for prokaryotes.

## Introduction

Photosynthetic light reactions are catalyzed by several protein complexes, namely Photosystem I (PSI), Photosystem II (PSII), cytochrome *b*_6_*f* complex, and ATPase synthase. The light energy needed to drive electron transfer is funneled to the photosystems by light-harvesting antenna complexes in cyanobacteria represented by Phycobilisomes (PBS). They are localized on the TM peripherally attached to the stromal side of PSI and PSII. It is well known that in higher plants photosystems are heterogeneously distributed with higher PSII content typically found in granal (stacked) thylakoids (Andersson and Anderson, 1980; Albertsson, 2001). Recently (Strašková et al., 2019), a heterogeneous distribution of the photosystems has also been identified in cyanobacteria *in vivo* without changes in membrane stacking. These heterogeneous TM areas were described as MDs (Konert et al., 2019; Strašková et al., 2019) and defined as membrane regions with different composition of pigment-proteins complexes (PPCs), it means photosystems and PBS. These special membrane zones, sub-micrometer in size, define the mosaic-like structure of TM. The MD structure is very stable in a span of minutes and it seems to restrict the overall mobility of all PPCs in cyanobacteria thylakoids (Strašková et al., 2019).

The importance of TM heterogeneity is still not fully clear (Mullineaux, 2005; Pribil et al., 2014). Several theories were proposed to explain its benefits for photosynthesis, such as: (1) reduction in PSI–PSII energy spillover; (2) solution of long-distance plastoquinone diffusion; (3) acting in fine-tuning of light-harvesting during photoprotection (Herbstova et al., 2012). The last point is in line with the fact that light intensity and/or its fluctuation is a key factor affecting overall photosynthetic efficiency. The periods with excessive light are potentially harmful to photosynthetic proteins, pigments, and lipids due to the formation of ROS (Li et al., 2009). Therefore, there are several photoprotective and light-optimizing processes (see, e.g., reviews for cyanobacteria; Kirilovsky et al., 2014; Calzadilla and Kirilovsky, 2020) that either dissipate excessive irradiation (non-photochemical quenching), regulate excitation energy distribution into/between photosystems (e.g., state transitions; McConnell et al., 2002; Kaňa et al., 2012; antenna decoupling; Kaňa et al., 2009; Tamary et al., 2012) or they cope with accelerated degradation of proteins in light (e.g., photoinhibition; Li et al., 2018). However, a functional link between the response of TM organization and fluctuations in light is still rather fragmented.

The granal/stromal organization of TMs in higher plants is affected by changes in growth light (Kirchhoff, 2013) or by light stress (Herbstova et al., 2012). In the case of cyanobacterial MDs, the effect of high or fluctuating light conditions on membrane organization is less clear. Here we decided to study the plasticity of TM microdomains during a shift from CL to L-D cycle. We also wanted to test whether there is a link between cell shape (regular, elongated, dividing, and string cells) and MD organization of TM. We proved that heterogeneity of thylakoids is not a simple consequence of cell’s phase in cell cycle; MDs are present in all types (shapes) of cells. Further, the MD structure is able to respond to a shift from CL to L-D conditions by more even redistribution of PSI in cells. Last but not least, cells kept their heterogeneity in sizes and shapes during the diel cycle that brought the discussion on importance of phenotypic heterogeneity in cyanobacteria.

## Materials and Methods

### Strain, Cultivation, Experimental Conditions

We used the PSI-YFP tagged strain (Strašková et al., 2018, 2019) of the glucose-tolerant *Synechocystis* sp. *PCC 6803* (hereafter *Synechocystis PSI-YFP*). Before cultivation in a bioreactor (FMT150, PSI, Brno, Czech Republic, see, e.g., Nedbal et al., 2008), cells were grown in an Erlenmeyer flask for 12 days under CL (fluorescence tubes 35 μmol m^–2^ s^–1^, 28°C, BG11 medium, continuous shaking) and regularly diluted to keep them in exponential growth phase. 400 mL of culture (OD_73__5_ = 0.4) was then diluted with 500 mL of BG11 and transferred into a bioreactor. Cells in the bioreactor were acclimated for 2 days to sinusoidal light without dark period and then to the sinusoidal L-D period (12/12 h, white light provided by diodes, maximal intensity of 100 μmol photons m^–2^ s^–1^, 28°C) for 2 weeks. The culture was regularly diluted to keep cells in the exponential growth phase.

For experiments in Erlenmeyer flasks, stock culture was initially cultivated under CL (for 3 days, fluorescence tubes) and then split into two cultures cultivated differently, either under continuous or sinusoidal L-D cycle for the following 10 days. Subsequently, the light conditions were switched (continuous to sinusoidal and vice versa) and kept for 7 days to follow the reversibility of thylakoid acclimation to each light condition.

### Cell Counter and Absorbance Measurements

Cell growth in the Erlenmeyer flasks was monitored by OD_73__5_ by WPA S800 spectrophotometer (Biochrom Ltd., England). The absorbance spectra of intact cells were measured by Unicam UV-500 (Thermo Spectronic, United States) by the integration sphere (Kaňa et al., 2009) and each data point represents an average of three daily samples (*n* = 3). Cell counts/sizes were estimated by Coulter Counter (Beckman, Multisizer 4, United States) at constant parameters (50 μL sample dilution in 10 mL of electrolyte made of 0.9% NaCl in deionized water; 50 μm aperture; size threshold level 1–4 μm) and averaged values were acquired by measuring three times per data point (*n* = 18). The curves of distribution in cell sizes were divided to get a percentage of small (range 1.2–1.6 μm) and large (range 1.601–2.5 μm) cells.

### 77 K Fluorescence Measurements

Low-temperature fluorescence (at 77 K) was recorded by SM-9000 (Photon Systems Instruments, Brno, Czechia) by averaging three emission spectra and repeated three times per day at different days in the bioreactor (*n* = 9). Parameters of measurements were as follows: excitation at 461 and 526 nm by LED; spectra detection from cells on GF-F filters (Whatman, United Kingdom); dark-adapted cells (20 min); baseline-correction with blank filter immersed with BG 11 medium.

### Confocal Microscopy and Image Processing

Images were acquired using Laser Scanning Microscope LSM 880 (Zeiss, Germany) using the Plan-Apochromat 63 × /1.4 Oil DIC M27 objective. Live *Synechocystis PSI-YFP* cells were imaged in three channels: (1) YFP from PSI-YFP (excitation 514 nm; detected 526–588 nm); (2) PBS (excited at 633 nm; detected at 642–677 nm); (3) Chl from PSII (excited at 488 nm; detected at 696–758 nm). The collected images (8 bit; 512 × 512; 8.24 μs dwell time; 1 Airy unit pinhole) were processed in ImageJ (FIJI distribution). First, the individual cells were cut from the whole pictures, and single-cell parameters were analyzed cell-by-cell. Each cell was characterized by its cell area and total fluorescence of each channel. Further, cells were categorized into one of the four types based on their shape (regular, elongated, dividing, string) in a similar way as described before (Schneider et al., 2007). The categories were selected automatically based on cell circularity (4π^∗^area/perimeter^2^), roundness [4^∗^area/(π^∗^major_axis^2^)], and feret diameter (the longest distance between any two points along the selection boundary). Category “regular” represented cells approaching circle (circularity > 0.8), “elongated” were ellipse-shaped (circularity < 0.8, roundness < 0.9, feret > 2.2), “dividing” were ellipse-shaped with constriction mark (circularity < 0.85, feret > 2.8). The string cells were constituted by two daughter cells after division (i.e., they were separated based on fluorescence pictures from thylakoids but connected based on transmission pictures) with circularity < 0.7. In required cases, some cells were manually re-assigned to a more fitting category. Total number of cells analyzed per day was between *n* = 709–1565 for bioreactor and *n* = 947 for Erlenmeyer flask experiments. In summary, about 10,000 pictures of *Synechocystis PSI-YFP* thylakoids were analyzed and interpreted.

### Statistical Analysis

The statistical analysis was carried out with R 3.6.2 (RCoreTeam, 2019) in Rstudio 1.2.5033 (RStudioTeam, 2019). Packages used: dplyr (Wickham et al., 2020), agricolae (de Mendiburu, 2020), and DescTools (Signorell et mult. al, 2020). Data were analyzed with Student’s *t*-test (Student., 1908) or one-way ANOVA (Fisher, 1921) when more than two data points were compared. Subsequent analysis with *post hoc* Duncan test (Duncan, 1955) was used when applicable. All significant points (*p* < 0.05) as compared with initial testing day were marked with asterisk on corresponding figures with colors matching the data being addressed.

## Results

**FIGURE 1:**
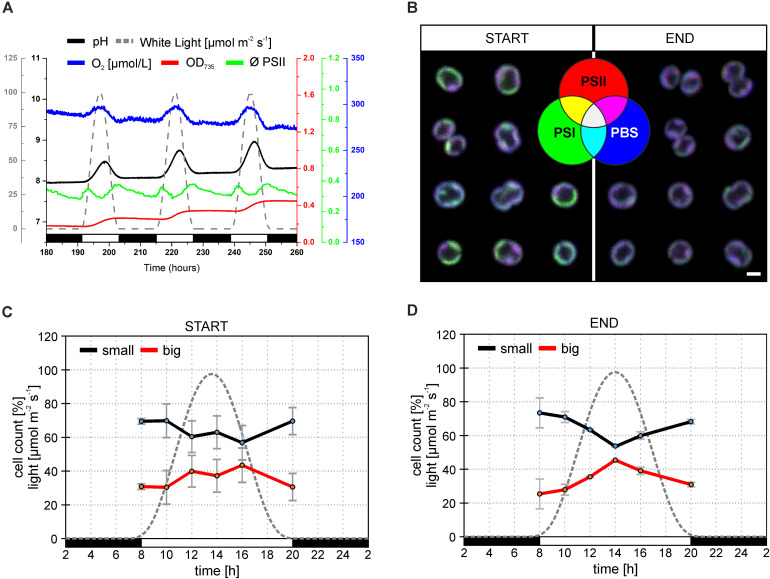
Behavior of *Synechocystis PSI-YFP* cells during cultivation in the bioreactor. The original starting culture (“START”; cells acclimated to continuous light; days 2 and 3 in the bioreactor) was slowly acclimated to the sinusoidal light-dark cycle (12/12 h marked by white/black bars, maximal intensity 100 μmol m^– 2^ s^– 1^). The light intensity profile is depicted as a gray dotted line. Cells grew in the bioreactor for 2 weeks to be considered as acclimated to the light-dark cycle (“END”; days 13 and 14 in the bioreactor). **(A)** Dynamic behavior of *Synechocystis PSI-YFP* cells during cultivation in the bioreactor *in situ.* Parameters represent: (1) OD_735_ optical density (red line) measured at 735 nm representing growth of biomass; (2) pH of culture (black line); (3) O_2_ dissolved in water (blue line); (4) ϕ_PSII_ (green line) representing actual PSII efficiency under red light; (5) light intensity (μmol m^– 2^ s^– 1^; gray dashed line). Data represent typical behavior of the parameters for days 6, 7, and 8 of bioreactor experiment. **(B)** Confocal microscope images of *Synechocystis PSI-YFP* cells at the start (days 2 and 3) and at the end (days 13 and 14) of bioreactor experiment. Three channel RGB pictures represent three acquired fluorescence signals showing localization of three complexes: (1) red—PSII autofluorescence; (2) blue—PBS autofluorescence; (3) green—PSI-YFP fluorescence. A combination of these three signals provides four additional membrane areas reflecting the co-localization of PSI/PSII/PBS. Overlapping signals create: (4) magenta—dominant PSI and PSII (low PBS); (5) cyan—dominant PSI and PBS (low PSII); (6) yellow—dominant PSI and PSII (low PBS); (7) white—PSI, PSII, and PBS are in similar, high content. Pictures represent typical thylakoid membrane organization of *Synechocystis PSI-YFP* during bioreactor experiment. Total number of analyzed cells per day was between *n* = 709 and 1565. **(C,D)** Confocal microscope measurements of cell sizes during the diel cycle, based on acquired images, two cell categories were counted: small (black line, cell area 2.0–2.8 μm^2^); big (red line, cell area 2.8–4.0 μm^2^). Data represent averages and SD at the start (**C**; days 2 and 3) and at the end (**D**; days 13 and 14) of the bioreactor experiment. Data represent averages of two different days of the bioreactor experiment.

*Synechocystis* sp. PCC 6803 strains with YFP tagged PSI (hereafter *Synechocystis PSI-YFP)* (Strašková et al., 2018, 2019) were first cultivated in CL condition and then shifted into sinusoidal L-D regime in the bioreactor to study the progressive acclimation of *Synechocystis PSI-YFP* to the more natural condition mimicing day-night cycle. The process was monitored *in situ* inside the bioreactor (Figure 1A). Cell growth was characterized by an increase in OD_735_ (Figure 1A); it indicted that cells grew only in light period and they stopped growth in the dark. This is in line with previous *in situ* observation with single cyanobacteria cell (Yu et al., 2017); it is obvious as *Synechocystis PSI-YFP* rely on light energy from photosynthesis. At maximal light irradiation, cell culture showed a depression in PSII maximal quantum yield (Φ_PSII_) typical for photoinhibition. Despite the high light induced effect in PSII fluorescence, the overall photosynthetic production of oxygen by PSII was not affected, as O_2_ concentration increased during daytime (Figure 1A). Photosynthetic activity was also followed by measuring pH of the growths medium in the bioreactor (Figure 1A). The culture pH can be used as an indirect measure of photosynthetic CO_2_ assimilation, because pH increases when the dissolved CO_2_ is removed from the water through photosynthesis. The pH (i.e., CO_2_ consumption) of the culture followed a similar pattern as the oxygen evolution (Figure 1A).

**FIGURE 2:**
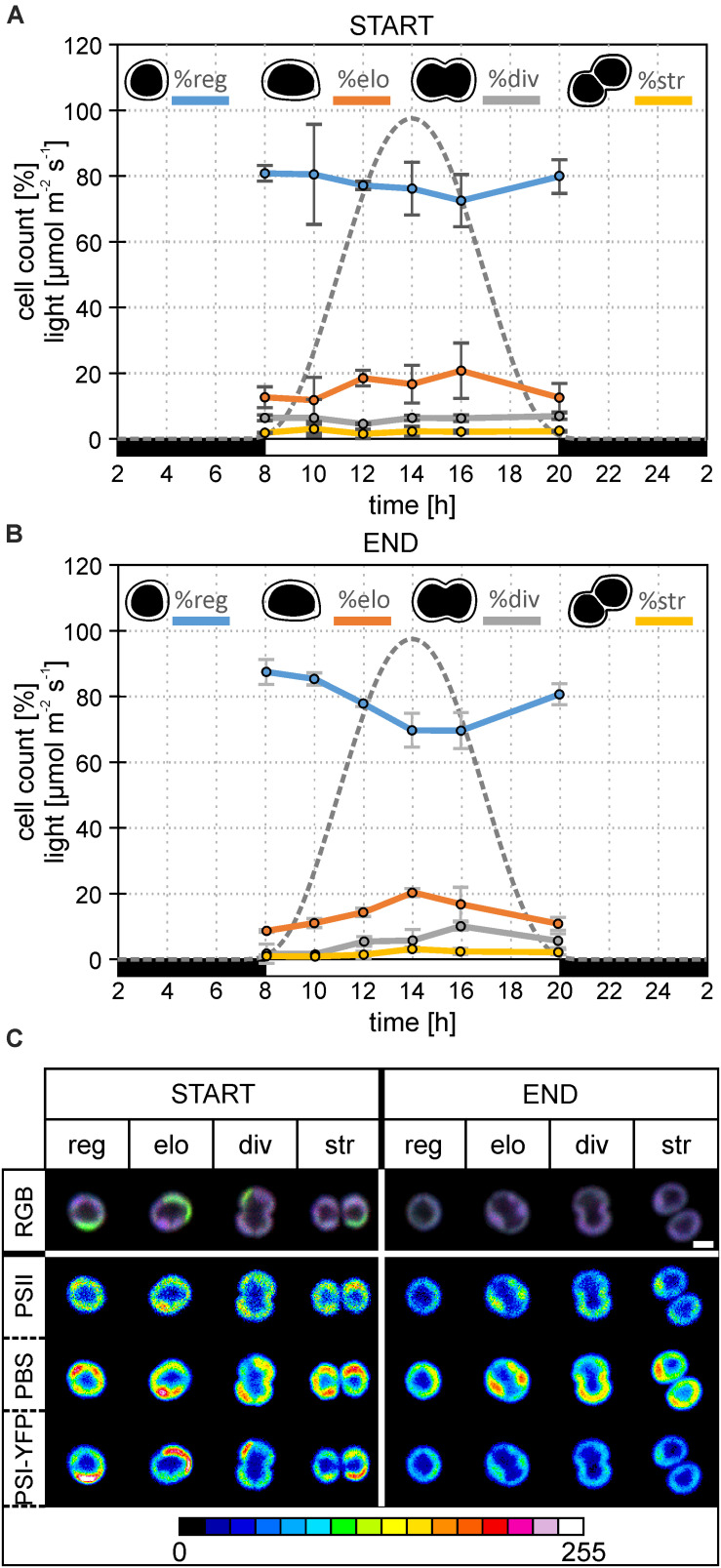
Changes in the diel distribution of cell types and microdomain localization in the particular type of *Synechocystis PSI-YFP* cells. The original starting culture (“START”; cells acclimated to continuous light; days 2 and 3 in the bioreactor) was slowly acclimated in the bioreactor to the sinusoidal light- night cycle (“END”, days 13 and 14 in bioreactor). The light intensity profile is shown as a gray dotted line. The figure describes diel changes in the cell counts and microdomains organization in the four cell types based on their shapes, namely: “*reg*”—regular cells; “*elo*”—elongated cells; “*div*”—dividing cells; “*str*”—string cells after division. Total number of cells analyzed per day was between *n* = 709 and 1565. **(A)** Diel cycle of relative cell counts of four typical cells—regular, elongated, dividing, string—the start of bioreactor experiment (i.e., *Synechocystis PSI–YFP* acclimated acclimated to continuous light). Intensity of sinusoidal light during the day is marked by a gray dotted line. Data represent averages and SD from 2 days of experiment (days 2 and 3). **(B)** Diel cycle of relative cell counts of four typical cells—regular, elongated, dividing, string—end of bioreactor experiment (i.e., *Synechocystis PSI*–*YFP* acclimated to light-dark cycle). Intensity of sinusoidal light during the day is marked by a gray dotted line. Data represent averages and SD from 2 days of the experiment (days 13 and 14). **(C)** A typical microdomain organization of four typical cells—regular, elongated, dividing, string. The figure compares the start (days 2 and 3) and at the end (days 13 and 14) of the bioreactor experiment. Total number of cells analyzed per day was between *n* = 709 and 1565. The first row shows three channels pictures (RGB, 24-bit) with co-localization of PSII, PBS, and PSI-YFP. Colors reflect PSI/PSII/PBS co-localization, the most dominant colors were magenta (dominant PBS and PSII), green (dominant PSI), white (balanced PSI, PSII, and PBS), and blue (dominant PBS). Second, third, and fourth rows depict intensity of single-channel fluorescence of PSII, PBS, and PSI-YFP, respectively. Colors reflect intensity of fluorescence signal per channel (heatmap images) in the 8-bit scale 0–255 (see the color scale bar).

*Synechocystis PSI-YFP* cells were collected from bioreactor on regular intervals and the TM structure of *Synechocystis PSI-YFP* cells was monitored by confocal microscope (Figure 1B). At the start of the experiment (when cells were still acclimated to CL), thylakoids had characteristic structure, color organization, and intensity as usual photosynthetic MDs in the culture grown under CL conditions (Konert et al., 2019; Strašková et al., 2019), the most typical set-up for laboratory experiments with cyanobacteria. MDs, as they were defined (Strašková et al., 2019), are characterized by typical PSI/PSII/PBS ratios that are reflected in their colors (in RGB color coding) and visible in single-cell images of TMs. Our RGB images thus showed the extent of PSI (green channel), PSII (red channel), and PBS (blue channel) co-localization (Figure 1B—START). These three channels images can be used also to identify different types of MDs (see color coding scheme of the seven possible MDs types in Figure 1B—green, red, blue, cyan, yellow, magenta, white as defined in Strašková et al., 2019) that are characterized by different ratio of PSI/PSII/PBS fluorescence. MDs were clearly visible in the RGB pictures at the start of the experiment (cells acclimated to CL) with most pronounced colors (MDs) being: (1) green with high PSI content (low PSII and PBS); (2) magenta with high PBS and PSII (low PSI); and (3) white with similarly high PSI, PSII, and PBS content (Figure 1B). On the contrary, MDs at the end of the experiment (cells acclimated to L-D cycle) became more “bluish” (Figure 2C—END), indicating a relative increase in PBS fluorescence in comparison to PSI-YFP or PSII emission.

This process of MD acclimation during the shift from CL to L-D growth was further explored. First, we characterized changes in cell sizes (Figures 1C,D and Supplementary Figure 1), and shapes (Figures 2A,B). Cell sizes were estimated on the level of single cells (Figures 1C,D) or cell suspension (cell counter, Supplementary Figures 1A,B) during the diel cycle consisting of 12 h dark and 12 h sinusoidal light (Figures 2A,B). Both methods brought the same conclusions: we observed an outline of the diel pattern in *Synechocystis PSI-YFP* cell sizes (Figures 1C,D and Supplementary Figures 1A,B). Smaller cell (with diameter *d* < 1.6 μm) counts were continuously decreasing until hours 14–16 of diel cycle. On the contrary, the larger cells (*d* > 1.6 μm) showed the opposite pattern (Figures 1C,D). There were no qualitative differences in the diel pattern between the start and the end (Figures 1C,D and Supplementary Figures 1A,B); changes were just more pronounced at the end of the experiment. Therefore, even though based on macroscopic parameters from bioreactor (Figure 1A), the *Synechocystis PSI-YFP* culture seems to be synchronized (which is in line with previous results; van Alphen and Hellingwerf, 2015), it remained heterogeneous on single-cell level (see cell sizes/cell shapes in Figures 1D, 2B). In fact, there was no particular point at which all cells would divide at one moment. *Synechocystis PSI-YFP* cells remained heterogeneous in their sizes during the diel cycle (Figures 1C,D). This is visible in the accumulation of elongating and dividing cells around hours 12–14 of the diel cycle (Figure 2B). Interestingly, the heterogeneity of cyanobacteria culture is also visible in the TM organization in our *Synechocystis PSI-YFP* (Figure 1C). Despite cells and TM heterogeneity, cells tend to keep some averaged parameters (bulk parameters) constant (e.g., equilibration of average sizes between the start and the end; Supplementary Figure 2). On the other hand, the huge cell-to-cell heterogeneity in *Synechocystis PSI-YFP* culture (Figure 2B) did not allow us to depict a clear diel pattern in the intensity of the 3 fluorescence channels per cell (Supplementary Figure 3) that reflected relative changes in PSI-YFP, PSII, and PBS fluorescence/concentration per single cell. We cannot exclude existence of such a dial pattern in single cell PSI-YFP, PSII, and PBS fluorescence as more experimental data are necessary to prove this hypothesis.

To cope with the inevitable heterogeneity of cyanobacteria cell sizes and its putative effect on thylakoid organization (Figures 1C,D), we tried to categorize cells based on their shapes (Figures 2A,B) and study their TM heterogeneity separately (Figure 2A). For this purpose, acquired images of *Synechocystis PSI-YFP* cells were grouped into four cell types based on their shapes reflecting their stage in the cell cycle, namely: regular, elongated, dividing, and string cells (see description in the legend of Figure 2). The diel profiles in the cell types (Figure 2A) were very similar with the diel profile of cells sizes (Figures 1C,D); we observed a continual decrease in the regular and increase in the number of elongated and dividing cells until midday (14 h in Figure 2B) when cells were more prone to cell division.

These four cell categories were then characterized by their MD organization (Figure 2C). At first, the heterogeneous structure of *Synechocystis PSI-YFP* thylakoids (defined by co-localization of PSI, PSII, and PBS; Figure 2A) was clearly visible for all four types of cells (Figure 2C). During the transition from CL to L-D cycle, all cell types become more “bluish” (RGB pictures in Figure 2C). This conclusion was confirmed by two additional independent bioreactor experiment (Supplementary Figure 4). In all cases, during the transition from CL to L-D cycle, cells have partially lost their green fluorescence signal due to the PSI-YFP decrease. On the other hand, the blue signal (reflecting PBS) was kept almost at the same levels between CL and L-D conditions (Supplementary Figure 4). It led to the more “bluish” cells thylakoids in all studied biological replicates (Figure 2C and Supplementary Figure 4A). When we analyzed the three measured channels independently in more details in the selected bioreactor experiment (see the heatmap in the Figure 2C), we clearly observed that the most pronounced spatial variability inside thylakoid was detected for PBS (high fluorescence red-spots in the heatmaps of PBS in Figure 2B). Moreover, its heterogeneity remained stable during CL to L-D transition in the bioreactor. On the contrary, the fluorescence signal of PSII (based on Chl autofluorescence) and even more PSI (based on YFP fluorescence) partially lost their heterogeneity during the CL to L-D switch (Figure 2B). This led us to conclude that the localization of PSII and especially PSI (in contrast to PBS) became more homogenous in thylakoids (Figure 2B) when cells are acclimated to the natural L-D cycle.

**FIGURE 3:**
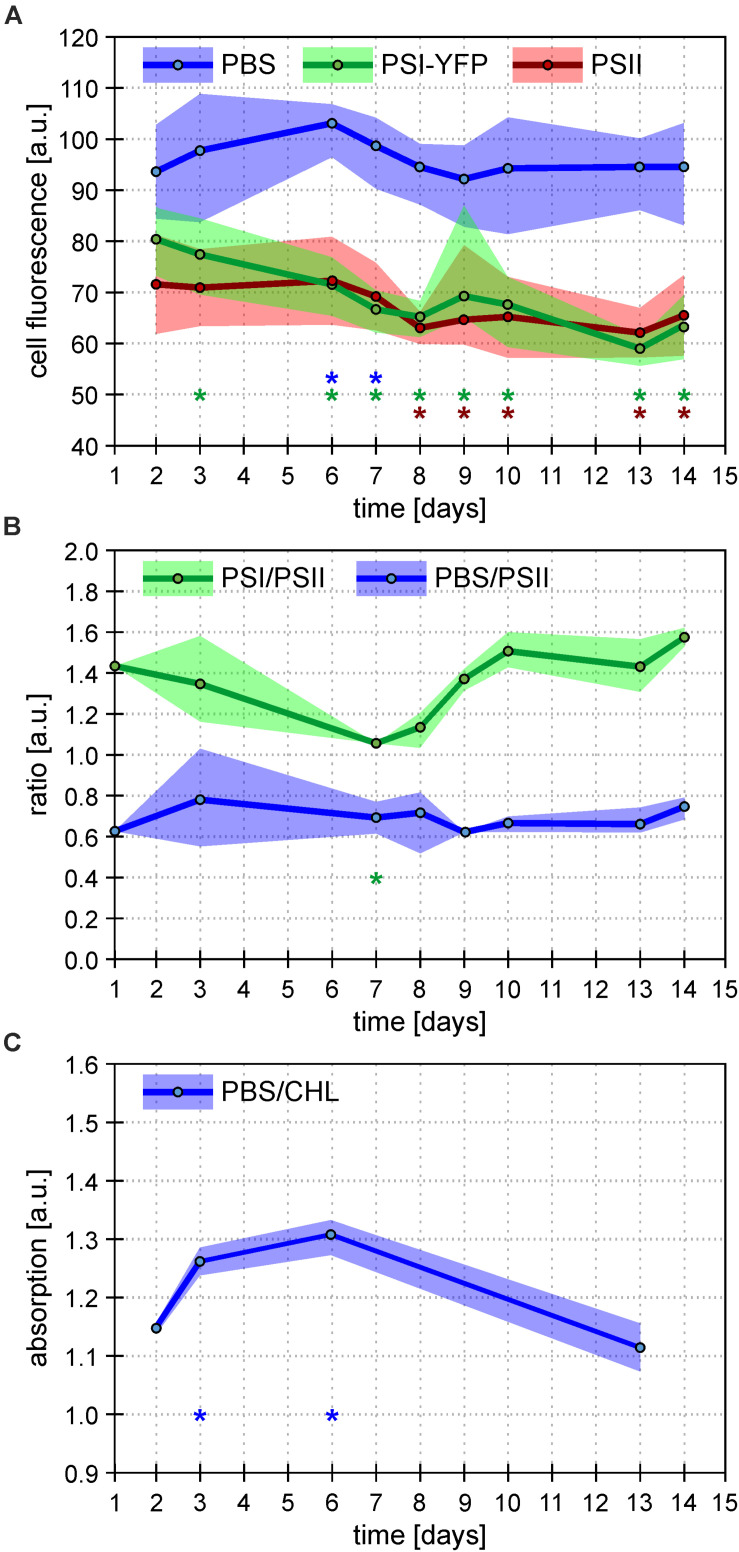
Overall behavior of *Synechocystis PSI-YFP* cells during cultivation in bioreactor. Figures depict continual acclimation of *Synechocystis PSI-YFP* cells after their shift from continuous light (day 0) to day–night cycle in bioreactor (days 1–14). **(A)** Intensity of single-cell fluorescence of pigment–protein complexes calculated from confocal images. Three channels were detected: PSI-YFP (green), PSII (red), and PBS (blue). The day values represent averages from six different time spots per day (see Supplementary Figure 3 for the whole diel cycle), ranges shows variability during the same day (i.e. maximal/minimal values in the day, not SD). Total number of cells analyzed per day was between *n* = 709 and 1565. Asterisks indicate data points significantly different from corresponding fluorescence value at day 2 (*p* < 0.05; blue = PBS; green = PSI-YFP; red = PSII). **(B)** Bulk measurements of PSI/PSII and PBS/PSII fluorescence ratios measured at 77 K. PSI/PSII fluorescence ratio (green line) reflects ratio of photosystems (excitation at 461 nm, detection at F730/F695 nm). The PBS/PSII fluorescence ratio (blue line) is a measure of PBS decoupling (excitation at 531 nm, detection at F660/F695 nm). Points represent averaged values per day (*n* = 9); ranges show variability during the same day (i.e. maximal/minimal values in the day, not SD). For whole spectra, see Supplementary Figure 5. Asterisks indicate data points significantly different from corresponding ratio value at day 2 (*p* < 0.05; green = PSI/PSII; blue = PBS/PSI). **(C)** Bulk measurements of absorbance ratio (623/630 nm) reflecting ratio of phycobilisomes to chlorophylls from photosystems (PBS/CHL). Points represent averaged values of the selected days (*n* = 3); ranges show variability (i.e. maximal/minimal values in the day, not SD) during the same day. Asterisks indicate data points significantly different from corresponding absorption value at day 2 (*p* < 0.05).

The process of this acclimation was quantified by calculating single-cell fluorescence intensity of PSI-YFP, PSII, and PBS (Figure 3A). The analysis showed a cell diel pattern in the three channels’ fluorescence; an initial decrease in all three channels’ fluorescence, and its recovery in the second half of the light phase of the day (Supplementary Figure 3). Further, the averaged values per every measured day (Figure 3A) clearly proved that PBS fluorescence emitted by single cell was the most stable during the 2 weeks acclimation under L-D cycle; through a slight increase in the first week was leveled down at the end to recover to the original value. On the contrary, PSI-YFP and PSII fluorescence continuously went down by about 20 and 10%, respectively (Figure 3A) indicating a decrease in PSI and PSII content or some change in fluorescence yield during acclimation from CL (start) to L-D cycle (end).

To further explore if the effect is either due to some changes in concentration of PPCs or due to a decrease in their fluorescence quantum yield, we estimated independently PSI/PSII ratio by 77 K fluorescence emission (Figure 3B). The bulk measurement of PSI/PSII ratio showed an initial decrease during the first 7 days (Figure 3B), which correlated with a much faster decrease of PSI fluorescence visible on single cells level (Figure 3A). Later, the confocal data from single cells (Figure 3A) and PSI/PSII ratio from bulk measurements (Figure 3B) behaved slightly differently. We do not know the precise mechanism behind this. It could be caused by a different change in quantum yield of PSI and PSII fluorescence in re-organized MDs due to some activation state transitions or/and some other de-quenching mechanism.

In comparison to photosystems, behavior of PBS fluorescence per cells was totally different during transition from CL to L-D cycle; we have noticed surprising stability of PBS fluorescence between initial and final values (Figure 3A). This result was compared with several bulk 77 K fluorescence and RT absorbance measurements. At first, fluorescence ratios of F650/F695 reflecting PBS decoupling (PBS/PSII in Figure 3B) has been detected (Figure 3B and Supplementary Figure 5). Further, the ratio of PBS to total Chls was deduced from the absorbance spectra (A_630/_A_682_) (PBS/CHL; Figure 3C). In the first 7 days, we found a temporal stimulation of PBS decoupling (day 3 in Figure 3B and Supplementary Figure 5) together with temporal increase in the PBS content reflected by PBS/CHL (day 3 in Figure 3C). The increase correlated with temporal stimulation in PBS fluorescence per single cell (days 3 and 6 in Figure 3A). However, all these three temporal effects disappeared after full CL to L-D acclimation (day 14 in Figures 3A–C) that shows the final stability of PBS fluorescence per cell (Figure 3A). The stability in PBS highly contrasted with the behavior of photosystems since their fluorescence decreased during CL to L-D transition (Figure 3A). Taking all this together, single *Synechocystis PSI-YFP* cell was more stable considering PBS fluorescence as it showed the same value during CL (days 1 and 2 in bioreactor) and L-D cell (days 13 and 14 in Figure 3). On the other hand, photosystems in the membrane were highly re-structured during CL to L-D transition; this is visible on single cell level as a more homogenous PSI distribution inside the cell (Figure 2C and Supplementary Figure 4). All these data shows that MDs are naturally occurring structures of TM (Strašková et al., 2019) and that they show plasticity in response to light conditions.

**FIGURE 4:**
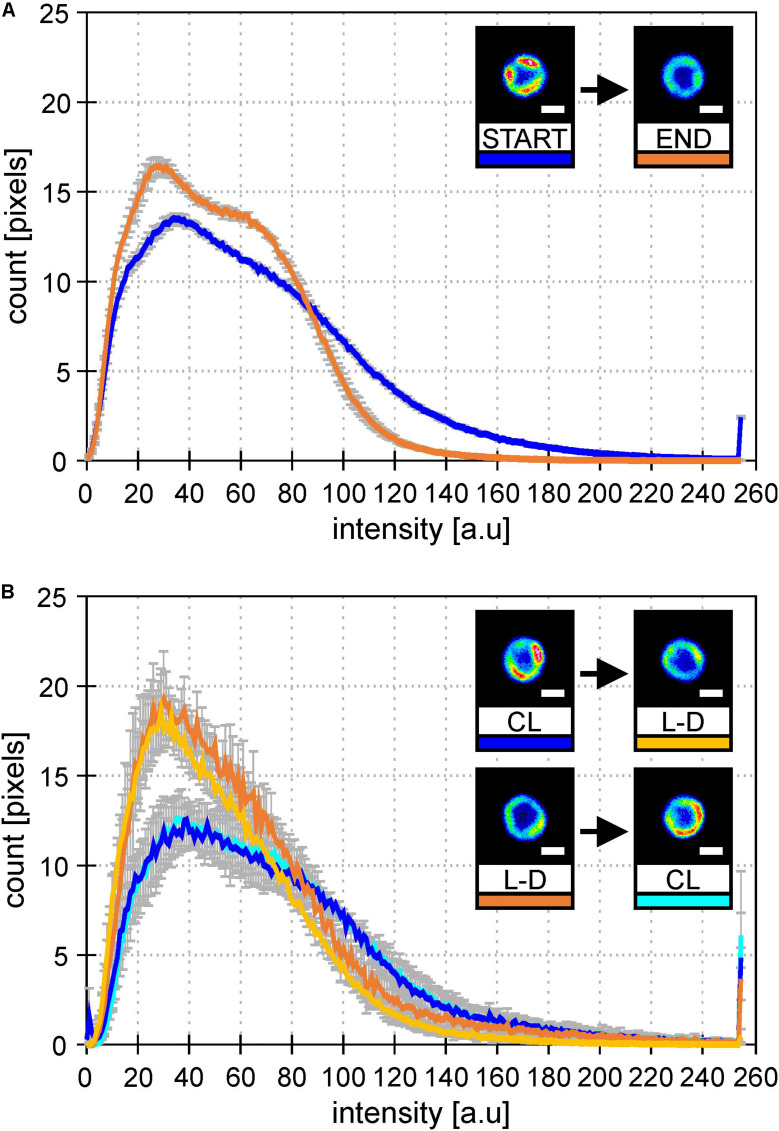
Histograms of PSI-YFP fluorescence intensities from thylakoids of *Synechocystis PSI-YFP* cells. Figures describe distribution of the PSI-YFP fluorescence intensity in cells grown either on continuous light (“CL”) or at light-dark cycle (“L-D”) in bioreactor and in flask. Histograms represent intensity profiles obtained from 615 **(A)** and 4276 **(B)** cells. Scale bars in the pictures show 1 μm. Total number of cells analyzed per day was *n* = 947. **(A)** Bioreactor data: Figures depicts distribution of PSI-YFP fluorescence intensities at the start (blue; days 2 and 3) and the end (orange; days 13 and 14) of bioreactor experiment. The pictures show typical PSI-YFP thylakoid distribution in cells from the START (cells acclimated to continuous light) and the END (cells acclimated to day–night cycle for 2 weeks) of bioreactor experiment. **(B)** Erlanmeyer flask experiment: Plots of PSI-YFP fluorescence intensity distribution for cells grown in the flask at continuous of light-dark cycle. Histograms and figures describes two experiments: (1) cells grown for 10 days on the continous light (“CL”; blue) were moved into the light-dark condition for 10 days (“L-D”; yellow); (2) cells grown in the light-dark condition (“L-D”; orange) for 10 days were moved into continous light (“CL”; cyan).

To check whether it was L-D cycle and not bioreactor itself causing this MD behavior, we established similar growth conditions in Erlenmeyer flasks. Cultures were switched between CL and L-D and then back to original condition to see the reversibility of the process (Figure 4). In both types of cultivation (bioreactor data in Figure 4A and flask data in Figure 4B), the addition of dark period caused spatial homogenization of PSI-YFP distribution visible in the cell pictures. Further, there was also clear narrowing of the PSI-YFP fluorescence in histograms pointing to the same effect (Figures 4A,B). The acclimation from growth without (CL) and with (L-D) dark period was clearly reversible as seen in the switch between CL and L-D for flask experiment (Figure 4B). It seems that *Synechocystis PSI-YFP* cells responded to the addition of a dark period into continuous irradiation by reorganizing PSI into more homogeneous pattern visible at natural L-D cycle (Figure 3).

## Discussion

We have identified the ability of cyanobacterial TM to acclimate to the switch in light condition by long-term reorganization of photosystems, especially PSI. Except the TM plasticity, we also observed MDs diversity in population of cells in line with previous results (Konert et al., 2019; Strašková et al., 2019). We have raised a hypothesis that the qualitative switch in PSI organization (more homogeneous PSI distribution during L-D than during CL irradiation; Figure 2C and Supplementary Figure 4) is triggered by the addition of a dark period for L-D cycle. The hypothesis has been proved in the flask experiments when light conditions were switched between CL and L-D and back to the original conditions. The variability in PSI distribution (more homogeneous in L-D, more heterogeneous in CL) and reversibility of the process (Figure 4B) shows the plasticity of cyanobacterial MDs. Even though MDs are very stable during short term changes in irradiation (e.g., in minutes; Strašková et al., 2019), some previous data have already indicated process of long-term acclimation in scale of hour(s) on single cell level (Steinbach and Kaňa, 2016) or on cell population level (Konert et al., 2019). Here we show clear response in PSI re-distribution after a long-term change in growth irradiation from CL to L-D conditions.

Our data indicate that the key factor in the acclamation to L-D cycle seems to be the redistribution of photosystems, especially PSI (Figure 2C). The special PSI redistribution could be helpful to better cope with diurnal changes in metabolism of cyanobacteria at diel cycles (Welkie et al., 2019). In fact, during the L-D cycles, there is an everyday shift from daytime photosynthesis to night-time oxidative pentose phosphate pathway (Diamond et al., 2015). The night-time is also used for cell detoxification from ROS. It is a question, whether the special redistribution of PSI at L-D cycle is favorable for larger NADPH production (formed in photosynthesis) that is preferentially used (more than NADH) as a reductant source for ROS detoxification (Flores and Herrero, 2005). The similar light-induced response on PSI level is visible also for cyanobacterial cells acclimated to high-light (Kopečná et al., 2012) or to a light of different quality (El Bissati and Kirilovsky, 2001; Luimstra et al., 2020). It is well know that a shift from low-light to high-light growth conditions stimulates decrease in the PSI to PSII ratio due to selective suppression of the amount of functional PSI (Murakami and Fujita, 1993; Murakami et al., 1997). Another important light-induced regulatory mechanism connected with PSI is light induced state transitions (see, e.g., Kirilovsky et al., 2014 for review). Interestingly, one from the older model of state transition proposed also PSI monomerization (Bald et al., 1996) and changes in spatial organization photosystems in TM during state transitions. In that model, PSII particles are aligned in rows in state 1 compared to state 2 with more randomly distributed particles (Olive et al., 1986, 1997). In light with the above-mentioned results, we tend to suggest that also TM plasticity, as we saw it based on PSI/PSII and PBS co-localization (Figure 2), is controlled by PSI to PSII ratio. The change in the ratio is induced by light and includes regulation of several genes (Luimstra et al., 2020). However, it is still not clear whether PSI to PSII ratio is a redox-control (El Bissati and Kirilovsky, 2001) or a photoreceptor control process. It needs to be still clarified whether TM plasticity connected with PSI redistribution (see Figure 2) is a redox or a photoreceptor control mechanism.

Redox/photoreceptor control is the only well-known control mechanisms of TM plasticity (Baulina, 2012). It needs to include various membrane-connected phenomena from the ion-induced effect on TM electrical double layer (Kaňa and Govindjee., 2016) to some changes in membrane energization (effect of DCMU in Stingaciu et al., 2019). In addition to photosystems, also PBS could be another factor in play, as TM morphology is changed if PBS structure is affected by mutation (Olive et al., 1997; Collins et al., 2012). Indeed, we have found a temporal appearance of the PBS decoupling in the middle of our 2 weeks experiment (Figures 3B,C and Supplementary Figure 5). The actual importance of PBS decoupling for cyanobacteria physiology and photoprotection is still matter of discussion (see, e.g., Kirilovsky et al., 2014; Calzadilla and Kirilovsky, 2020 for reviews). Several works have been published proposing physiological importance of PBS decoupling at various circumstances including high light stress (Tamary et al., 2012; Steinbach and Kaňa, 2016) or state transitions (Kaňa et al., 2009; Kupper et al., 2009; Chukhutsina et al., 2015; Ranjbar Choubeh et al., 2018). Our data indicate that PBS decoupling could play another role during reorganization of TM proteins. In a situation when photosystems need to be slowly redistributed in thylakoid during few days, PBS are more prone to be decoupled (Figures 3B,C) and it can be detected by a typical increase in the PBS emission on single cell level (Figure 3A) or in the whole suspension (Supplementary Figure 5). As soon as photosystems are reorganized in a new type of a steady distribution (i.e., thylakoids are acclimated to L-D cycle), the PBS fluorescence disappears as the proper PSI–PBS and PSII–PBS interactions are re-established. Indeed, the increase in the PBS fluorescence disappeared on the end of bioreactor experiment, when cells were acclimated to the L-D cycle (Supplementary Figure 5). Surprisingly, when comparing the first and the last day during transition from CL to L-D, the PBS composition (deduced from a single cell fluorescence) seems to be unaffected; PBS were then more stable then photosystems (Figures 3B,C). Therefore, the increase in the PBS decoupling was only a temporal process that seems to be conditional for successful reorganization of photosystems in TM during changing light conditions.

Our bioreactor experiment showed structural changes in organization of TM proteins within hours/days. Similar short-term structural plasticity of TM (in minutes) is also known as light/dark-induced TM swelling/shrinking visible in electron micrograph (Murakami and Packer, 1970). This process has been recently confirmed by neutron scattering method (Nagy et al., 2011; Stingaciu et al., 2019). However, this effect is probably undetectable by our method as MDs seem to be very stable in short term (Strašková et al., 2019). Recently, there were other studies proposing the fast reorganization of TM proteins during few minutes of very high light (Sarcina et al., 2006; Tamary et al., 2012; Casella et al., 2017). These data, though, do not correspond with the observed stability of MDs (Strašková et al., 2019). The observed discrepancy could be a result of rather non-physiological high irradiances used in these studies (from tens to hundred thousand μmol m^–2^ s^–1^). Other explanation could be that such dynamic behavior can be present only in few specific cells (see discussion of phenotypic heterogeneity below). In fact, we agree with these authors that cyanobacterial TM proteins have the ability to be reorganized in thylakoids based on current light conditions. However, our data show rather slower kinetics behind (hours-days). The organization of pigment-proteins in MDs is rather stable in minutes of physiological high-light (Strašková et al., 2019), that is also supported by their very low mobility (see recent reviews, Kirchhoff, 2008; Mullineaux, 2008; Kaňa, 2013). Based on our data, reorganization of TM requires hours (Steinbach and Kaňa, 2016; Konert et al., 2019) or days (Figures 2C, 4A,B) to be clearly visible and detectable on single cell level as different types of MDs. Therefore, instead of the term “dynamics of TM,” we prefer to talk about “TM plasticity” in a similar meaning as it is known for higher plants thylakoids (Pribil et al., 2014). Indeed, our cyanobacterial MDs are able to be reorganized in a similar way as it can be seen for granal/stromal thylakoids (Kirchhoff, 2013).

Our data pointed out that TM plasticity needs to be considered in light of cell to cell heterogeneity of cyanobacteria. We provide evidence of *Synechocystis PSI-YFP* population heterogeneity considering their sizes (Figure 1) and shapes (Figure 2) during the diel cycle. The phenomenon has been already noted by the previous work (Strašková et al., 2019) where count of MDs per single cell was shown to be variable (from one to four). It indicates that research on cyanobacterial response to variable light conditions requires single cell methods because cyanobacterial cells are not uniformed. It seems that such population heterogeneity in cyanobacteria cultures seems to be inevitable as cyanobacteria cells often forms two subpopulations (Martins et al., 2018). It is caused by multiple factors that coordinate cyanobacterial cell growth and division (Yang et al., 2010). The main factors for most of bacteria could be listed as environmental (e.g., light conditions for photothrophs), the internal circadian clock, and the cell-volume control (Nordholt et al., 2020). Those three interacts also in cyanobacteria in a complex manner and form heterogeneous population of cells. For instance, considering volume control, cyanobacteria behave as “adders”—they are prone to divide when a certain volume has been added after division (Yu et al., 2017). Interestingly, it is in contrast to current view on other phototrophs (algae), where the cell cycle progression is considered to be regulated by critical size and algae can be fully synchronized naturally by alternating light/dark period (Nishihama and Kohchi, 2013). Various groups have shown that the cell division and growth in algae are tightly linked to light levels (Bišová and Zachleder, 2014) in contrast to cyanobacteria (Yang et al., 2010). In this prokaryotic photothrophs, cell division is freely “allowed” at certain times of the day and the division window can only be narrowed by light/day cycles (Martins et al., 2018).

Stepping from the single cell level to the cell population level, the phenomenon we observed in single cells, structural plasticity of thylakoid MDs, could also manifest a more general trend typical for bacteria cells: phenotypic heterogeneity (Grote et al., 2015). It describes the inevitable occurrence of “non-conformist” cells (cells with distinct phenotype) within isogenic (cyano)-bacterial populations. The phenotypic heterogeneity allows genotypes to persist in a fluctuating environment (Ackermann, 2015) like our variable L-D cycle. However, we still do not know in details why cyanobacteria keep their MD organization heterogeneous in the population (Konert et al., 2019; Strašková et al., 2019). The plasticity of MDs could be a manifestation of phenotypic heterogeneity, a recently broadly discussed phenomenon of non-genetic cell-to-cell differences in microbial population (Van Boxtel et al., 2017). More experimental work needs to be done to address the connection between the behavior of MDs structure on the single-cell and on the population level.

## Data Availability Statement

The original contributions presented in the study are included in the article/Supplementary Material. Further inquiries can be directed to the corresponding author/s.

## Author Contributions

RK designed and supervised the project and wrote the manuscript. MC and GK were involved in the project discussion, carried out all the experimental work, and analyzed the data. GK contributed to confocal microscopy. MC contributed to all other methods. All authors interpreted the data, discussed the results, and commented on the manuscript.

## Conflict of Interest

The authors declare that the research was conducted in the absence of any commercial or financial relationships that could be construed as a potential conflict of interest.

## Abbreviations

Chl: chlorophyll
CL: continuous light
d: cell diameter in μ m
L-D: light-dark cycle
MD(s): microdomain(s)
OD_735_: optical density measured at 735 nm
PBS: phycobilisomes
PPCs: pigment–proteins complexes (namely, PSI, PSII and PBS)
PSI: photosystem I
Φ _PSII_: maximal quantum yield of photosystem II in light or actual PSII efficiency in light
PSI: Photosystem I
PSII: photosystem II
ROS: reactive oxygen species
TM(s): thylakoid membrane(s).
