# Supplementary figures and images for: Plasticity of Cyanobacterial Thylakoid Microdomains Under Variable Light Conditions
Source: Front Plant Sci. 2020 Nov 12;11:586543. doi: 10.3389/fpls.2020.586543 (PMC7693714; doi:10.3389/fpls.2020.586543)

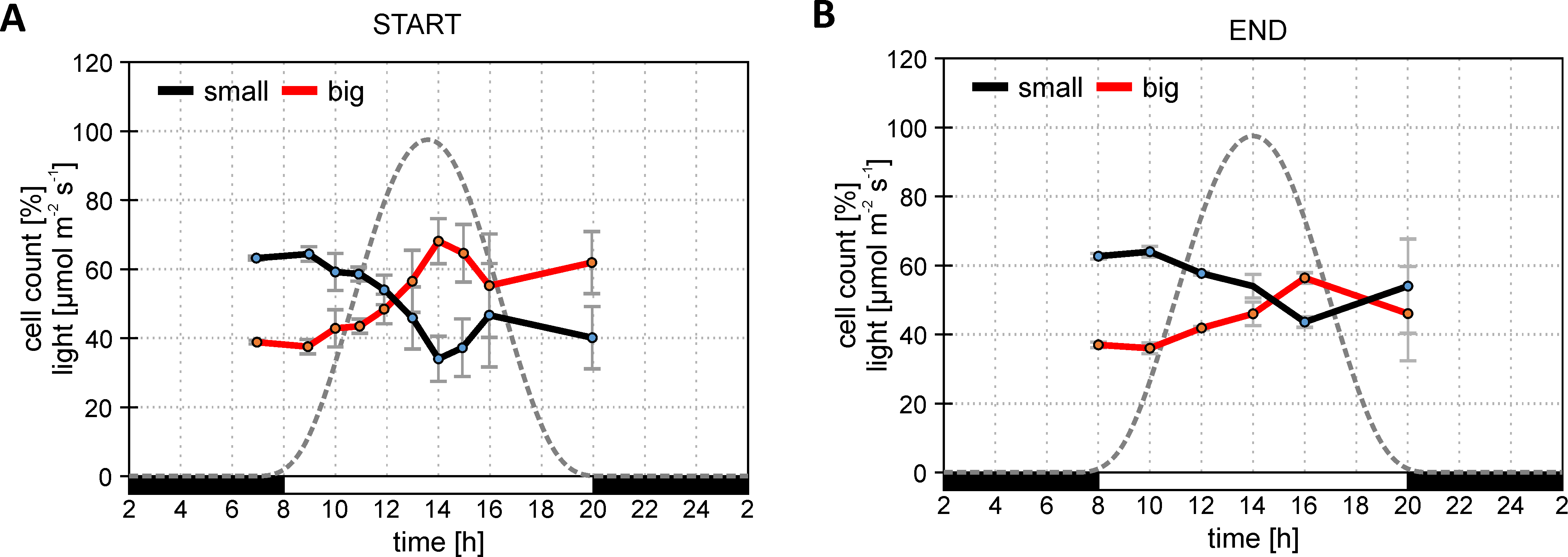

Supplement: Supplementary file 1 [file Image_1.tif]

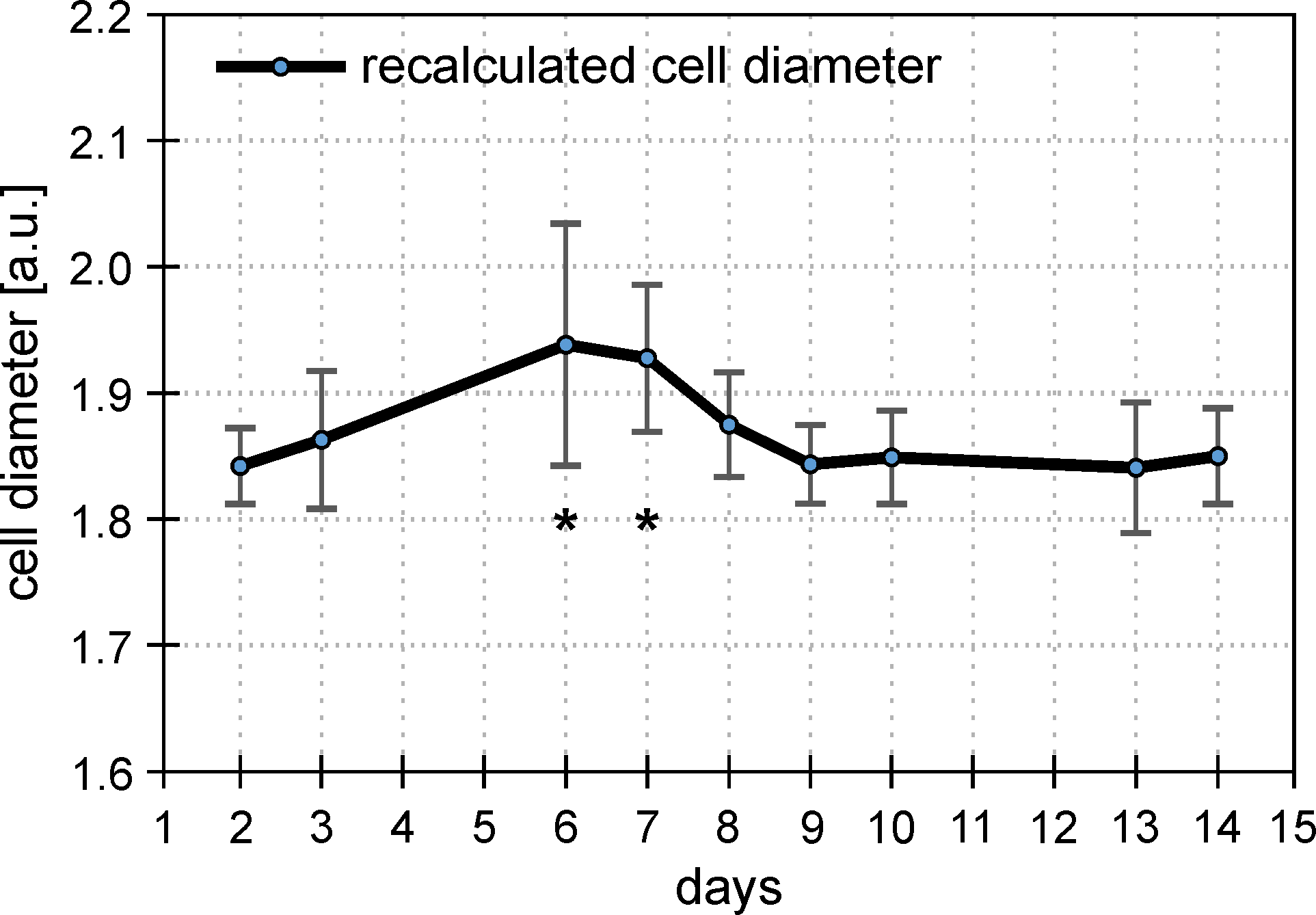

Supplement: Supplementary file 2 [file Image_2.tif]

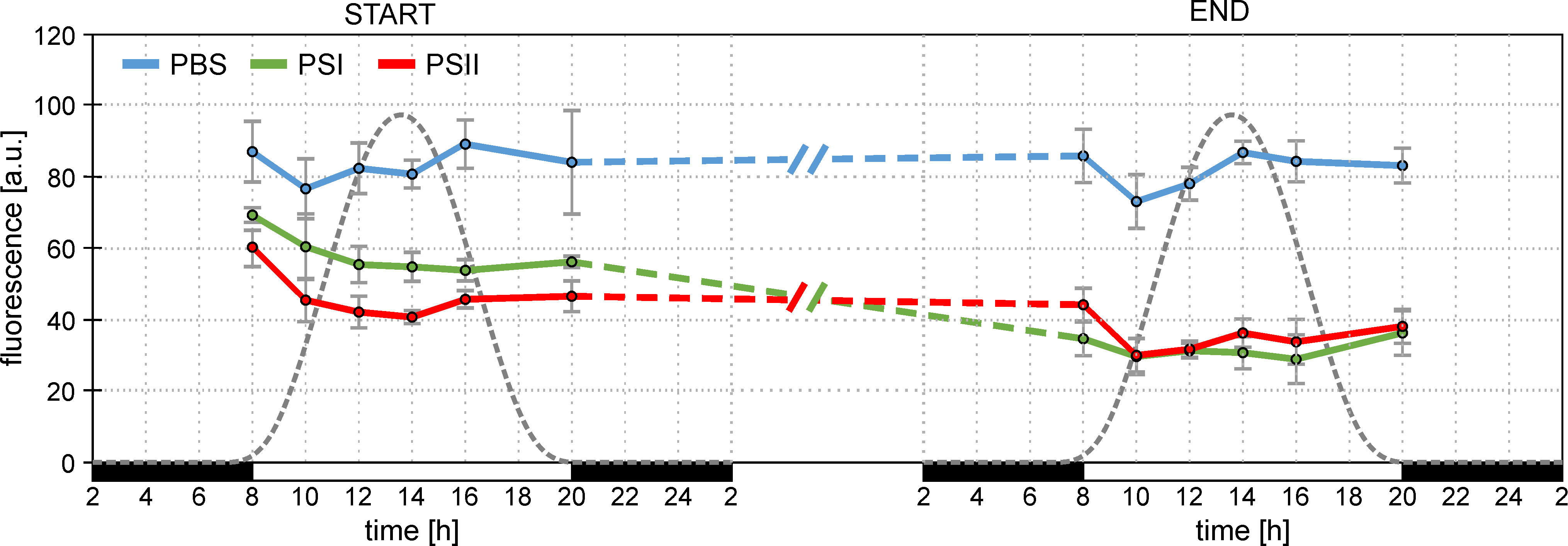

Supplement: Supplementary file 3 [file Image_3.tif]

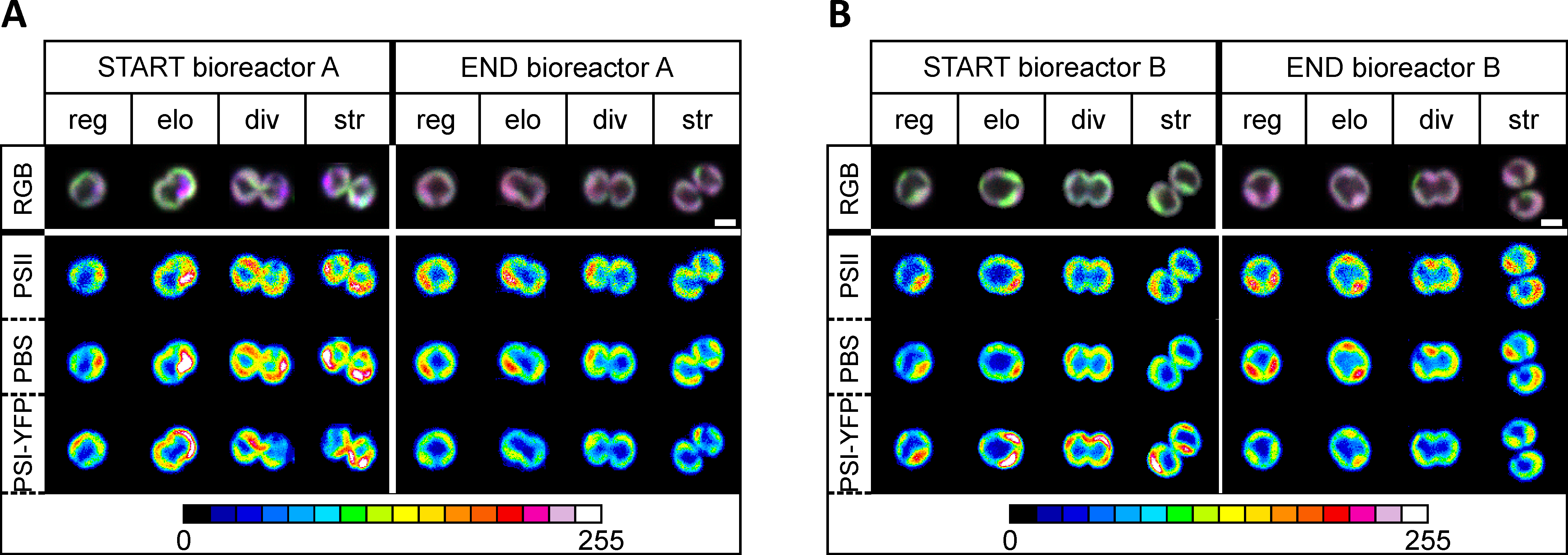

Supplement: Supplementary file 4 [file Image_4.tif]
