# Supplementary material for: Plasticity of Cyanobacterial Thylakoid Microdomains Under Variable Light Conditions
Source: Front Plant Sci. 2020 Nov 12;11:586543. doi: 10.3389/fpls.2020.586543 (PMC7693714; doi:10.3389/fpls.2020.586543)
Supplement: Supplementary file 6 [file Presentation_1.pdf]

SUPPLEMENTARY FIGURES

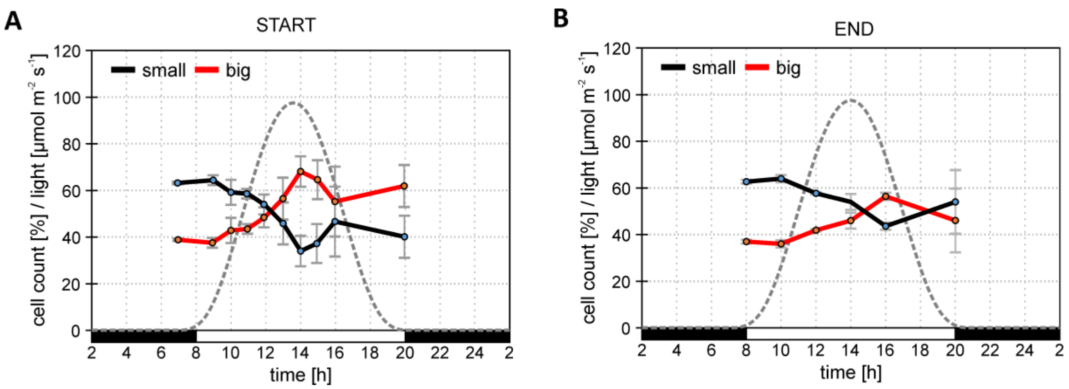

SUPPLEMENTARY FIGURE 1. Diel distribution of *Synechocystis PSI-YFP* sizes measured by cell counter. Cells sizes were measured by cell counter (n=18) and two categories of cells from bioreactor were counted from the cell counter histogram: small (black line, cell diameter 1.2-1.6 µm), big (red line, cell diameter 1.6-2.5 µm). Data represents averages and SD at the start (cell acclimated on continuous light; days 2 and 3) and at the end (cell acclimated on light-dark cycle; days 15 and 16) of the bioreactor experiment.

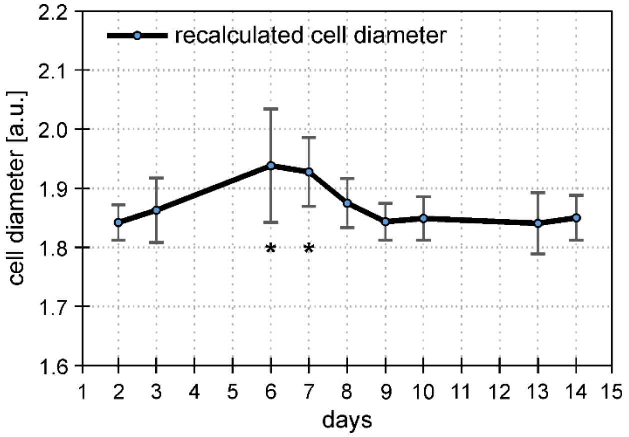

SUPPLEMENTARY FIGURE 2. Size of *Synechocystis PSI-YFP* cells during bioreactor experiment. Cells areas (A) obtained from image analysis was recalculated from all cells types and diameters (d) was then re-calculated to approximate round shape of cells ($d = 2 * \sqrt{A/\pi}$). Total number of cells analyzed per day was between n = 709 to 1565. Asterisk show data points significantly different from day 2 (p<0.05).

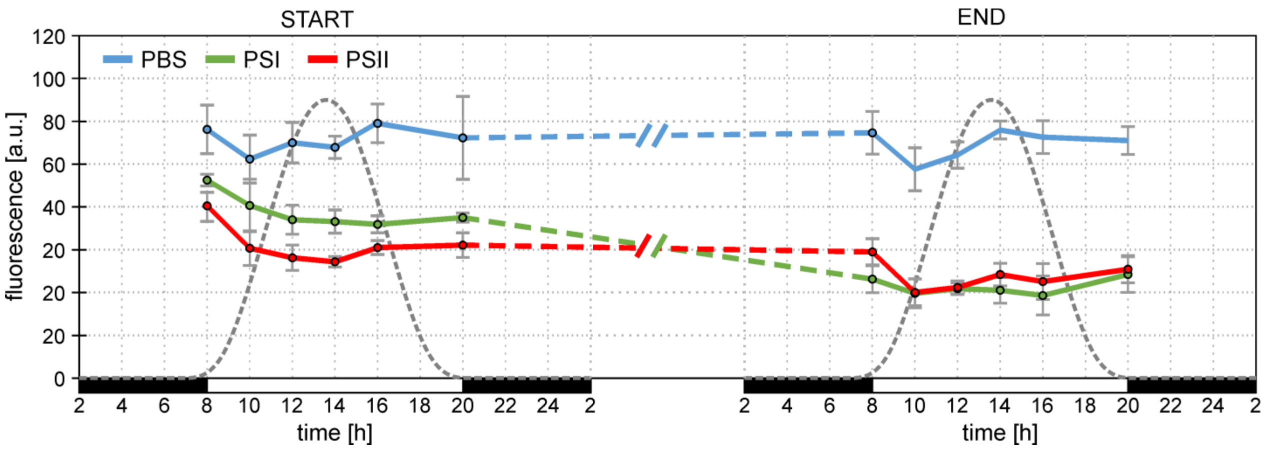

SUPPLEMENTARY FIGURE 3. Diel profiles of single-cell fluorescence of pigment-protein complexes calculated from confocal images of *Synechocystis PSI-YFP* cells from bioreactor. 3 channels were detected: PSI-YFP (green), PSII (red), and PBS (blue) at particular hours of light period of the day (8, 10, 12, 14, 16, 20 h). The fluorescence intensities were obtained from the whole cell fluorescence cell acclimated on continuous light at the START (days 2 and 3) and on the END (days 13 and 14) of bioreactor experiment when cells were acclimated on light-dark cycle. The day averaged values are presented in the Figure 3A including day-maximal and day-minimal values as plotted ranges. Light/night periods of the day are marked by white/dark bars, light intensity profiles profile as grey dotted line. Total number of cells analyzed was between n = 709 to 1565.

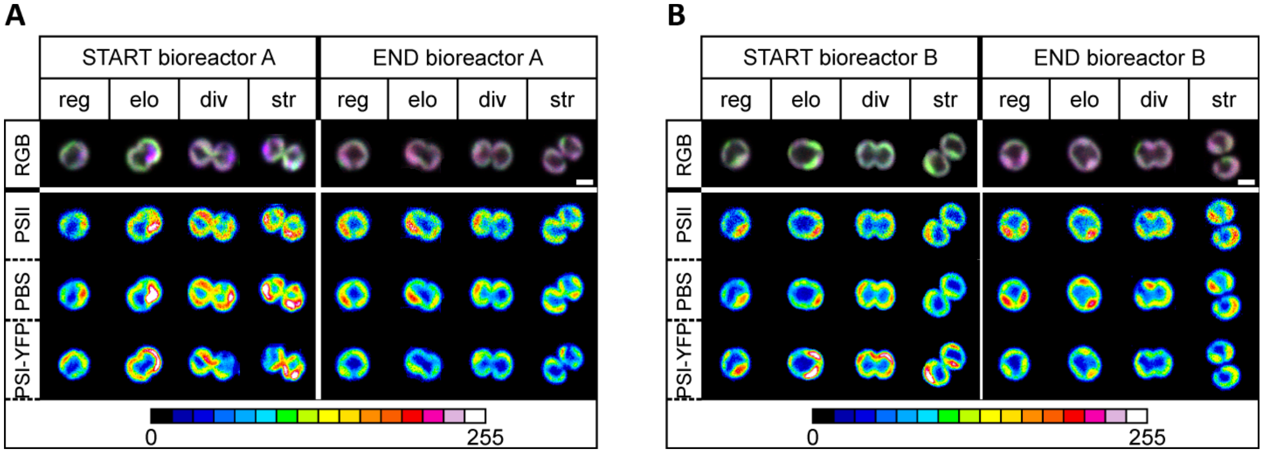

SUPPLEMENTARY FIGURE 4. Changes in the microdomains composition during 2 weeks of bioreactor experiments. 2 additional independent experiments (biological replications A, B) are presented. Typical microdomain organization of 4 cells category – regular, elongated, dividing, string. The first row shows 3 channels pictures (RGB, 24-bit) with co-localization of PSII, PBS, and PSIYFP. Colors reflect PSI/PSII/PBS co-localization, the most dominant colors were magenta (dominant PBS and PSII), green (dominant PSI), white (balanced PSI, PSII, and PBS), and blue (dominant PBS). Second, third, and fourth rows depict intensity of single-channel fluorescence of PSII, PBS, and PSI-YFP respectively. Colors reflect intensity of fluorescence signal per channel (heatmap images) in the 8-bit scale 0-255 (see the color scale bar).

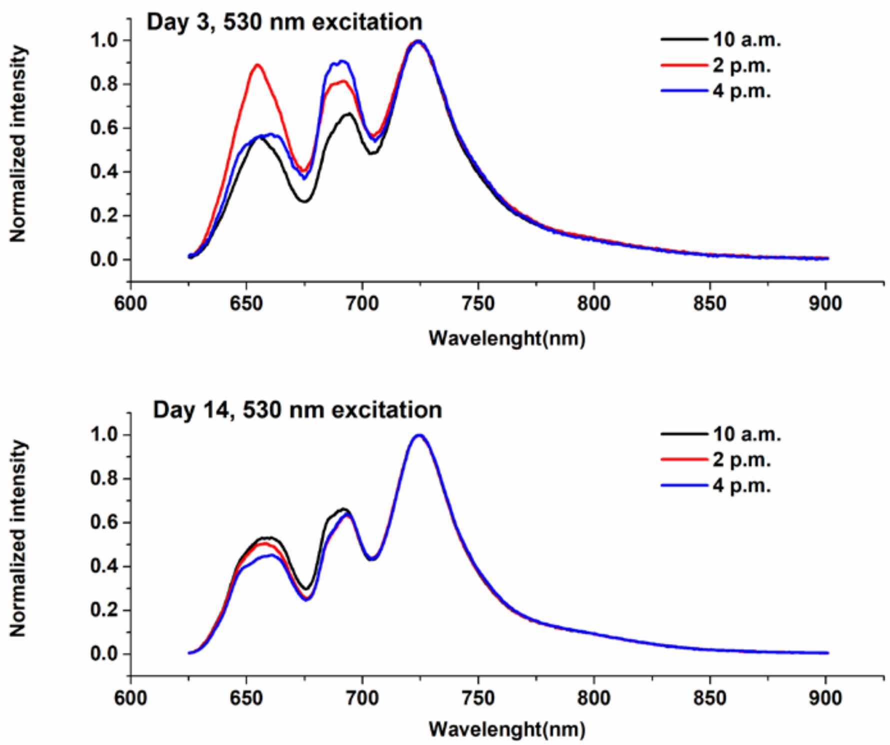

SUPPLEMENTARY FIGURE 5. Typical 77K spectra fluorescence spectra from *Synechocystis PSI-YFP* cells. Fluorescence was excited at 531 nm and spectra (n=9) were normalized to PSI fluorescence at F730 nm. The fluorescence maxims F660 (from PBS) and F695 (from PSII) were then used to calculate F660/ F6950 ratio presented in the Figure 3B. The spectra were collected at the particular day-time (10 a.m., 2 p.m., 4 p.m.) for light dark profile used in bioreactor experiment (sinusoidal light intensity profile, light periods starting at 8 a.m., maximum light at 2 p.m., light off 20 p.m., see light profile in the Figure 1C and D).
